# The molecular mechanism of propionate-regulating gluconeogenesis in bovine hepatocytes

**DOI:** 10.5713/ab.23.0061

**Published:** 2023-06-26

**Authors:** Rui Pang, Xiao Xiao, Tiantian Mao, Jiajia Yu, Li Huang, Wei Xu, Yu Li, Wen Zhu

**Affiliations:** 1 College of Animal Science and Technology, Anhui Agricultural University, Hefei 230036, China

**Keywords:** Bovine, Gluconeogenesis, Hepatocytes, Propionate

## Abstract

**Objective:**

Cows that are nursing get around 80% of their glucose from liver gluconeogenesis. Propionate, a significant precursor of liver gluconeogenesis, can regulate the key genes involved in hepatic gluconeogenesis expression, but its precise effects on the activity of enzymes have not yet been fully elucidated. Therefore, the aim of this study was to investigate the effects of propionate on the activity, gene expression, and protein abundance of the key enzymes involved in the gluconeogenesis of dairy cow hepatocytes.

**Methods:**

The hepatocytes were cultured and treated with various concentrations of sodium propionate (0, 1.25, 2.50, 3.75, and 5.00 m*M*) for 12 h. Glucose content in the culture media was determined by an enzymatic coloring method. The activities of gluconeogenesis related enzymes were determined by enzyme linked immunosorbent assay kits, and the levels of gene expression and protein abundance of the enzymes were detected by real-time quantitative polymerase chain reaction and Western blot, respectively.

**Results:**

Propionate supplementation considerably increased the amount of glucose in the culture medium compared to the control (p<0.05); while there was no discernible difference among the various treatment concentrations (p>0.05). The activities of cytoplasmic phosphoenolpyruvate carboxylase (PEPCK1), mitochondrial phosphoenolpyruvate carboxylase (PEPCK2), pyruvate carboxylase (PC), and glucose-6-phosphatase (G6PC) were increased with the addition of 2.50 and 3.75 m*M* propionate; the gene expressions and protein abundances of PEPCK1, PEPCK2, PC, and G6PC were increased by 3.75 m*M* propionate addition.

**Conclusion:**

Propionate encouraged glucose synthesis in bovine hepatocytes, and 3.75 m*M* propionate directly increased the activities, gene expressions and protein abundances of PC, PEPCK1, PEPCK2, and G6PC in bovine hepatocytes, providing a theoretical basis of propionate-regulating gluconeogenesis in bovine hepatocytes.

## INTRODUCTION

During lactation, the liver and mammary gland perform complementary metabolic tasks, and the liver synthesizes glucose and releases it into circulation for absorption by the mammary gland [1]. Liver gluconeogenesis provides ruminants with 80% glucose, which plays an important role in the energy metabolism of ruminant [2]. Propionate is the primary precursor of gluconeogenesis among the substrates, and it can produce 50% to 60% of the glucose in a cow’s hepatocyte [3]. Oba et al [4] reported that the function of hepatic gluconeogenesis in ruminants was positively correlated with the supply of propionic acid.

There are several key enzymes in the gluconeogenesis pathway, such as glucose-6-phosphatase (G6PC), cytoplasmic phosphoenolpyruvate carboxylase (PEPCK1), mitochondrial phosphoenolpyruvate carboxylase (PEPCK2), and pyruvate carboxylase (PC). PC is crucial for accelerating the transformation of pyruvate into oxaloacetate, and the capacity of ruminants for gluconeogenesis is directly affected by PC expression [5]. Oxaloacetate-dependent GTP conversion to phosphoenolpyruvate is catalyzed by PEPCK [6]. The G6PC enzyme converts glucose-6-phosphate to glucose [7]. The activity and expression of these enzymes or genes may directly or indirectly affect the liver gluconeogenesis of ruminants [8].

Propionate has been shown to regulate the mRNA expression of genes associated with gluconeogenesis in bovine hepatocytes [9,10]. However, the directly effect of propionate on enzyme activity is still unknown. Therefore, the purpose of this experiment was to investigate the effects of propionate on glucose production, and the activity, gene expression and protein abundance of the enzymes involved in the gluconeogenic pathway of bovine hepatocytes.

## MATERIALS AND METHODS

### Animal care

The Anhui Agricultural University Ethics Committee for Animal Care and Use approved the use of animals (SYXK (Wan) 2016-007).

### Culture of bovine hepatocytes

The method of culturing bovine hepatocytes has been described in previous studies [11,12]. Briefly, the donors were healthy female calves purchased from a commercial dairy farm (Hefei, China). Calves were stunned and left decubitus by pentobarbital sodium (0.1 mL/kg body weight). An incision of about 20 cm was made in the right abdomen to cut through the skin, muscle layer and peritoneum. A retractor is placed inside the incision. The caudate lobe of the liver is obtained using a scalpel. Following perfusion cleansing of the obtained tissue, a two-step collagenase IV infusion was used to break down the caudate lobe [13]. After filtering, the suspension was centrifuged at 500×g for 5 min at 4°C. The cells were then revived in Dulbecco’s modified eagle medium (DMEM) with low glucose (Biosharp, Beijing, China) supplemented with 10% fetal bovine serum (Zeta Life, Menlo Park, CA, USA), 1 nM dexamethasone, 10 μg/mL vitamin C, and 1% antibiotic solution (Invitrogen, Carlsbad, CA, USA; streptomycin, 5×penicillin, amphotericin). The media was changed to DMEM with 10% fetal bovine serum and 1% antibiotic solution following 4 h of culture.

In 6-well plates, 1×10^6^ cells were seeded in each well for 44 h. For the next 12 h, DMEM media containing 0, 1.25, 2.50, 3.75, and 5.00 m*M* sodium propionate were employed.

### Glucose and enzyme activity determination

The media of the cell culture was collected after sodium propionate incubation, an enzymatic colored glucose oxidase kit was used to determine the glucose content in the culture media (cat. A154-1-1, Nanjing Jiancheng Bioengineering Institute, Nanjing, China). The enzyme activity of PEPCK1, PEPCK2, PC, and G6PC were determined using enzyme linked immunosorbent assay kits (catalog numbers ml001620, ml001621, ml001623, and cats. ml001629, respectively; Shanghai Enzyme-linked Biotechnology Co., Ltd., Shanghai, China).

### Gene expression assay

Using Trizol reagent (Takara, Dalian, China), total RNA was isolated from cow hepatocytes. The absorbance of A260 nm/A280 nm was then measured using the nucleic acid concentration detector (within 1.8 to 2.0). The integrity of the RNA was evaluated by electrophoresis on a 1% gel. Real-time quantitative polymerase chain reaction (PCR) was used to measure the relative mRNA abundance using the SYBR Green technique (Takara, China) and the ABI 7500 instrument (Applied Biosystems, Singapore). The primers used were listed in Table 1. The procedures of the PCR were used according to Zhang et al [10]. The relative expression of the genes was normalized to reference gene of *β*-actin in the same sample, and were calculated using the 2^−ΔΔCT^ approach (cycle threshold, CT) [14].

### Western blot analysis

Protein concentration was assessed using a bicinchoninic acid kit (Applygen Technologies Inc., Beijing, China) according to the method described by Li et al [15]. Western blot was performed according to our previous report [16]. Briefly, the protein sample was combined with the loading buffer, then heated in a 95°C water bath for 10 min. Protein samples (25 μg) were separated by 10% sodium dodecyl sulfate-polyacryl amide gel electrophoresis protein over 2 h at 80 v, and transferred to 0.45 μm polyvinylidene fluoride membranes (Millipore Corp., Billerica, MA, USA). After blocking with 5% (w/v) skim milk, the membranes were incubated overnight at 4°C with the primary antibodies: PEPCK1 (1:2,000; Bioss Biotechnology Co., Ltd, Beijing, China), PEPCK2 (1:2,000; Bioss, China), G6PC (1:2,000, Bioss, China), PC (1:1,000, Ab-mart, Biomedical Co., Ltd, Shanghai, China), and *β*-actin (Bioss, China). After washing for 5 min in Tris buffered saline with Tween 20, the membranes were incubated goat anti-rabbit secondary antibodies (Sangon, Shanghai, China) for 1 h. Finally, the reaction strips were evaluated using Bio-Rad software (version 5.2.1). The gray values of the target proteins and the *β*-actin were analyzed using Image J software. Then the gray values of the target protein were divided by the gray values of the *β*-actin and normalized.

### Statistical analyses

Data are expressed as the mean±standard deviation. One-way analysis of variance, followed by Duncan’s multiple range test in SPSS 22.0, was used for data analysis. A p-value<0.05 was considered statistically significant.

## RESULTS

### Propionate supplementation increased the glucose concentration

**Figure 1:**
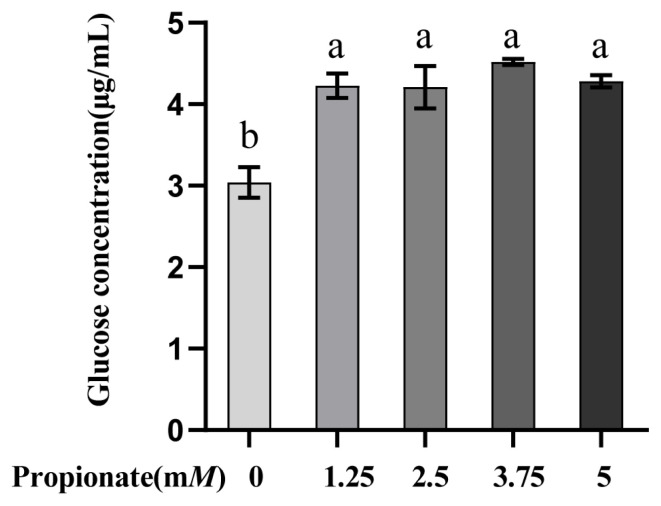
Effect of propionate supplementation (0, 1.25, 2.50, 3.75, 5 m*M*) on glucose concentration in the medium of bovine hepatocytes. The values are given as the mean±standard deviation (n = 3), and different letters ^a,b^ indicate significant differences (p<0.05).

As depicted in Figure 1. The addition of propionate increased the glucose concentration in comparison with the control (p<0.05), while there were no differences among the treatments (p>0.05).

### Propionate supplementation enhanced gluconeogenesis-related enzyme activity

**Figure 2:**
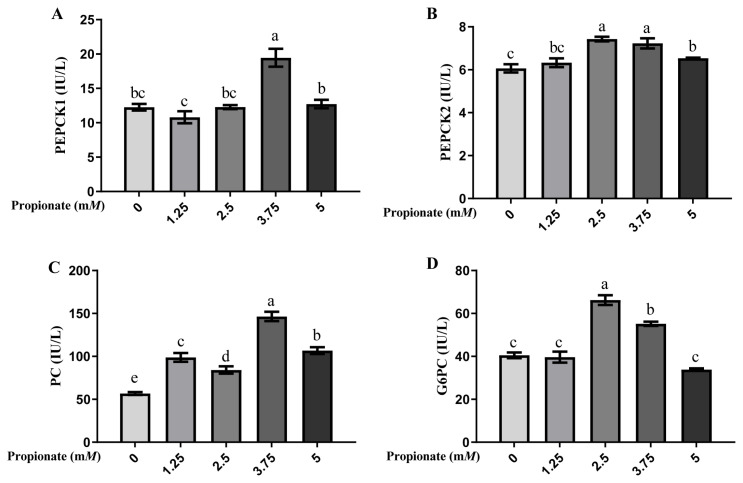
Effect of propionate supplementation (0, 1.25, 2.50, 3.75, 5 m*M*) on the enzyme activities of (A) cytosolic phosphoenolpyruvate carboxykinase (*PEPCK1*), (B) mitochondrial phosphoenolpyruvate carboxykinase (*PEPCK2*), (C) pyruvate carboxylase (*PC*), and (D) glucose-6-phosphotase (*G6PC*) in bovine hepatocytes. Values are the presented as mean±standard deviation (n = 3), and different letters ^a–e^ indicate significant differences (p<0.05).

As shown in Figure 2, the activity of PEPCK1 was greater in the bovine hepatocytes incubated with 3.75 m*M* propionate than with 0, 1.25, 2.50, and 5.00 m*M* propionate (p<0.05), with no difference among the other 4 groups (p>0.05). Compared with the control, the activity of PEPCK2 was induced by 2.50, 3.75, and 5.00 m*M* propionate (p<0.05), but no effect of propionate at 1.25 m*M* was noted (p>0.05). Furthermore, the activity of PEPCK2 was more effective with 2.50 and 3.75 m*M* propionate than with 5.00 m*M* propionate (p<0.05). The activity of PC was higher with propionate than that without propionate (p<0.05), and cells supplemented with 3.75 m*M* propionate enhanced the activity of PC compared with that at other 3 concentrations (p<0.05). Compared with the control, treatment with 2.50 and 3.75 m*M* propionate increased the activity of G6PC (p<0.05), whereas the activity of G6PC with 1.25 and 5.00 m*M* propionate did not show any changes (p>0.05).

### Propionate supplementation increased the mRNA expression of gluconeogenic-related enzymes

**Figure 3:**
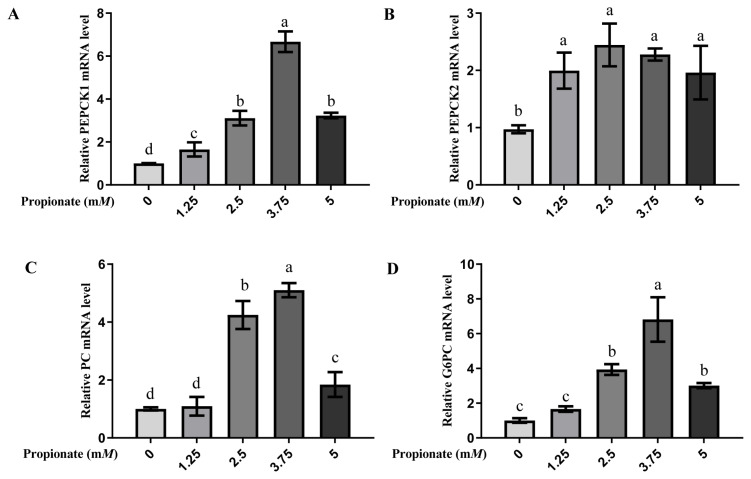
Effect of propionate supplementation (0, 1.25, 2.50, 3.75, 5 mM) on the mRNA expression of (A) cytosolic phosphoenolpyruvate carboxykinase (*PEPCK1*), (B) mitochondrial phosphoenolpyruvate carboxykinase (*PEPCK2*), (C) pyruvate carboxylase (PC), and (D) glucose-6-phosphotase (*G6PC*) in bovine hepatocytes. The mRNA levels of no propionate addition (0) were normalized to 1.0. Values are the presented as mean±standard deviation (n = 3), and different letters ^a–d^ indicate significant differences (p<0.05).

At all doses, the introduction of propionate increased the mRNA expression levels of PEPCK1 and PEPCK2 compared with no propionate (p<0.05; Figure 3). The mRNA expression level of PEPCK1 was greater in the bovine hepatocytes incubated with 3.75 m*M* propionate than with 0, 1.25, 2.50, and 5.00 m*M* propionate (p<0.05), while no effect on the mRNA expression of PEPCK2 with propionate among the treatment groups was noted (p>0.05). The expression of PC and G6PC were increased by addition of 2.50, 3.75, and 5.00 m*M* propionate compared with the absence of propionate (p<0.05), whereas there was no difference between the 0 m*M* propionate group and 5.00 m*M* propionate group (p> 0.05). Similarly, the addition of 3.75 m*M* propionate increased the mRNA expressions of both PC and G6PC compared to the other doses (p<0.05).

### Propionate supplementation increased the protein abundance of gluconeogenic-related enzymes

**Figure 4:**
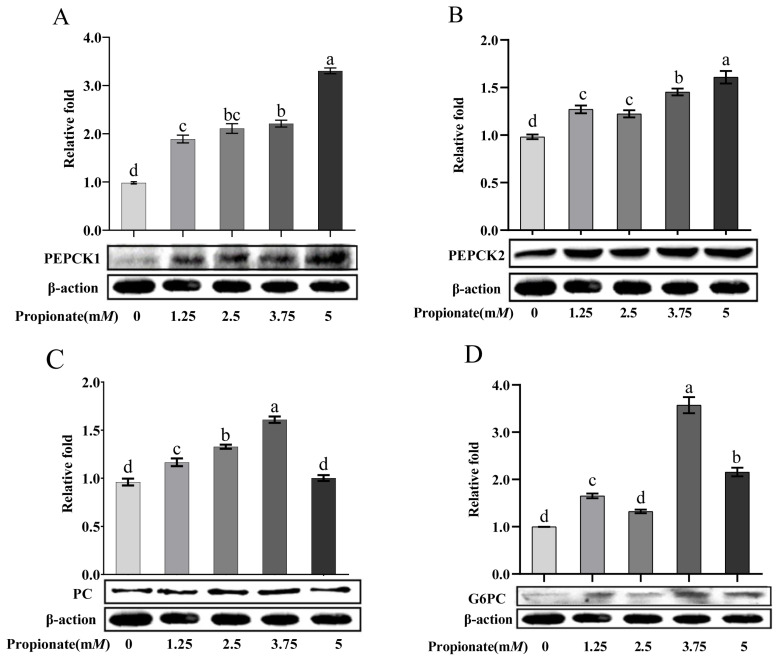
Effect of propionate supplementation (0, 1.25, 2.50, 3.75, 5 m*M*) on the protein abundance of (A) cytosolic phosphoenolpyruvate carboxykinase (*PEPCK1*), (B) mitochondrial phosphoenolpyruvate carboxykinase (*PEPCK2*), (C) pyruvate carboxylase (*PC*), and (D) glucose-6-phosphotase (*G6PC*) in bovine hepatocytes. Protein levels of no propionate addition (0) were normalized to 1.0. Values are the presented as mean± standard deviation (n = 3), and different letters ^a–d^ indicate significant differences (p<0.05).

As shown in Figure 4, the addition of propionate increased the protein abundance of PEPCK1 and PEPCK2 dose-dependently (p<0.05). Compared to the control, the protein abundance of PC was increased 2.50 and 3.75 m*M* propionate (p<0.05), but no effect of propionate at 5.00 m*M* was noted (p>0.05). Treatment with 1.25, 3.75, and 5.00 m*M* propionate increased the protein abundance of G6PC (p<0.05), whereas the abundance of G6PC with 2.50 mM did not show any changes (p>0.05). In particular, the protein abundances of PC and G6PC were higher in the bovine hepatocytes incubated with 3.75 m*M* propionate than with the other concentrations (p<0.05).

## DISCUSSION

Agarwal et al [17] reported that glucose entry and gluconeogenesis increased significantly after rumen infusion of sodium propionate or feeding diets supplemented with sodium propionate. We also found that propionate dramatically increased the content of glucose in the culture media, suggesting that propionate can encourage gluconeogenesis in bovine hepatocytes, which is consistent with previous research [18].

Key enzymes in the gluconeogenesis pathway include PC, PCK, and G6PC. As an important physiological regulator, PC can catalyze the carboxylation of ATP-dependent pyruvate using HCO_3_^−^ as a source of CO_2_, providing oxaloacetate required for gluconeogenic intermediates, which is then converted to phosphoenolpyruvate via the PEPCK enzyme [19]. In the current study, the activity, gene expression, and protein abundance were increased with propionate addition (2.50 and 3.75 m*M*), indicating an increased availability of oxaloacetate.

PEPCK enzymes are found in two distinct subtypes, one is in the cytoplasm and the other is in the mitochondria, with varying relative amounts in different species [20]. Oxaloacetic acid is transformed into phosphoenolpyruvate by PEPCK1, which is an essential step in cytoplasmic gluconeogenesis [21]. It has been suggested that the enzyme activity of PEPCK1 plays an important role in controlling the entry of propionate into the gluconeogenesis process [2]. Studies have reported that propionate stimulates the production of propionate gluconeogenesis and the mRNA expression of PEPCK1 in bovine hepatocytes [9,22]. We also showed that propionate supplementation increased PEPCK1 mRNA levels, with the greatest expression at 3.75 mM propionate, which is consistent with previous studies. Additionally, we measured the enzyme activity and protein abundance of PEPCK1, and the outcomes supported that PEPCK1 was regulated by propionate.

Oxaloacetate cannot directly enter the cytoplasmic matrix through the mitochondria. To facilitate gluconeogenesis, PEPCK2 converts oxaloacetate into phosphoenolpyruvate in the mitochondria and then enters the cytoplasmic matrix [21]. Studies on goat hepatocytes have revealed that the PEPCK2 enzyme is required for the conversion of most propionate to glucose in cells [23]. Propionate activates PEPCK2 mRNA in a dose-dependent manner in bovine hepatocytes [10]. Similar to earlier studies, this investigation discovered that propionate supplementation can increase the enzyme activity and mRNA expression of PEPCK2. It is interesting to note that the enzyme activity and protein abundance of PEPCK2 were lower than those of PEPCK1, demonstrating that the cytoplasm was the main place where oxaloacetate was converted to phosphoenolpyruvate.

The final step of gluconeogenesis, the hydrolysis of glucose-6-phosphate to produce glucose, is catalyzed by G6PC. One of the crucial enzymes for preserving blood glucose homeostasis is G6PC [24]. It has been reported that the mRNA expression of G6PC in bovine hepatocytes was unaffected by 2.50 m*M* propionate [10]. Additionally, according to Zhan et al [25], 3 m*M* propionate was also unable to alter the expression of G6PC mRNA in bovine intestinal epithelial cells. In our study, the mRNA expression of G6PC was not affected by propionate at a low concentration (1.25 m*M*), while was increased by propionate at higher concentrations (2.50 and 3.75 m*M*), indicating that the effect of propionate on G6PC mRNA expression was dose dependent.

## CONCLUSION

Our findings suggest that adding 3.75 m*M* propionate can enhance the enzyme activities, mRNA expressions, and protein abundance of PC, PEPCK1, PEPCK2, and G6PC, as well as further enhance the ability of bovine hepatocytes to produce glucose, providing a theoretical basis of propionate-regulating gluconeogenesis in bovine hepatocytes.

## Figures and Tables

**Table 1:** Primers for real-time polymerase chain reaction analyses

Gene	Accession number	Primer sequence (5′ to 3′)^1)^	Amplicon	Annealing temperature (°C)
*PEPCK1*	NM_174737.2	F:5-AGGGAAATAGCAGGCTCCAGGAAA-3 R:5-CACACGCATGTGCACACACACATA-3	175	60
*PEPCK2*	NM_001205594.1	F:5-TGACTGGGCAAGGGGAGCCG-3 R:5-GGGGCCACCCCAAAGAAGCC-3	412	60
*G6PC*	NM_001076124.2	F:5-AAAAGCCAACCTACAGATTTCG-3 R:5-TGAGCAGCAAGGTAGATTCG-3	102	60
*PC*	NM_177946.4	F:5-CCACGAGTTCTCCAACACCT-3 R:5-TTCTCCTCCAGCTCCTCGTA-3	108	60
*β-action*	NM_173979.3	F:5-TGTGCTGTCCCTGTATGCCTCTG-3 R:5-TTGGGAATGCTCGATCCAACCG-3	910	60

*PEPCK*1, cytosolic phosphoenolpyruvate carboxykinase; *PEPCK2*, mitochondrial phosphoenolpyruvate carboxykinase; *G6PC*, glucose-6-phosphatase; *PC*, pyruvate carboxylase.

1) F, forward; R, reverse.
